# 706. An Ad Hoc Subgroup Analysis of a Phase 3, Open-Label Study Indicates Efficacy and Safety of Fecal Microbiota, Live-jslm in Participants With Recurrent *Clostridioides difficile* Infection and Renal Impairment

**DOI:** 10.1093/ofid/ofad500.768

**Published:** 2023-11-27

**Authors:** Monika Fischer, Joan Thul, Beth Guthmueller, Christian Sandrock, Nicholas Van Hise, Glenn S Tillotson

**Affiliations:** Indiana University, Indianapolis, Indiana; Ferring Pharmaceuticals, Inc., Parsippany, New Jersey; Rebiotix Inc., a Ferring Company, Roseville, Minnesota; UC Davis School of Medicine, Sacramento, CA; Metro Infectious Disease Consultants, Burr Ridge, Illinois; GST Micro LLC, NORTH, Virginia

## Abstract

**Background:**

Patients with renal comorbidity are at risk of severe *Clostridioides difficile* infection (CDI) and recurrence. Fecal microbiota, live-jslm (REBYOTA™, abbreviated here as RBL, previously known as RBX2660) is the first FDA-approved, microbiota-based live biotherapeutic for the prevention of recurrent CDI (rCDI) in adults following antibiotic treatment for rCDI. An ad hoc subgroup analysis of PUNCH CD3-OLS (NCT03931941), an ongoing, open-label, phase 3 trial evaluating the efficacy and safety of RBL, assessed outcomes in participants with renal comorbidity.

**Methods:**

PUNCH CD3-OLS participants were ≥ 18 years old with medically documented rCDI, including first recurrence determined by the treating physician, and assessed with standard-of-care (SOC) diagnostic methods. After SOC antibiotics, participants received a single dose of rectally administered RBL. Treatment success was defined as remaining recurrence free for 8 weeks after treatment. Treatment-emergent adverse events (TEAEs) through 6 months of treatment were reported. Participants with renal comorbidity were identified from the medical history dictionary-derived terms in the modified intent-to-treat (mITT) population.

**Results:**

Within the mITT population, 98 of 402 participants with adjudicated outcomes had renal comorbidity, including chronic kidney disease (n=29) and end-stage renal failure (n=5). Of participants with renal comorbidity, 50% had Charlson Comorbidity Index scores of ≥ 5, versus 18% of participants without renal comorbidity. Treatment success was achieved by 66% (65/98) and 77% (235/304) of participants with and without renal comorbidity, respectively. TEAEs occurred in 71% (n=70) of participants with renal comorbidity and 64% (n=194) of participants without renal comorbidity. In both groups, most TEAEs were moderate in severity and related to preexisting conditions. Serious TEAEs were reported by 16% (n=16) and 8% (n=24) of participants with and without renal comorbidity, respectively. The most commonly reported serious TEAE was CDI recurrence, occurring in 3.1% (n=3) and 1.6% (n=5) of participants with and without renal comorbidity, respectively.

**Conclusion:**

RBL treatment success and TEAE incidence were numerically comparable for those with and without renal comorbidity.

**Disclosures:**

**Monika Fischer, MD**, Ferring Pharmaceuticals Inc.: Advisor/Consultant|Rebiotix Inc., a Ferring Company: Board Member|Seres Pharmaceuticals: Advisor/Consultant **Joan Thul, BA**, Ferring Pharmaceuticals Inc.: Employee **Beth Guthmueller, AS**, Rebiotix Inc., a Ferring Company: Employee **Christian Sandrock, MD**, Allergan: Advisor/Consultant|National Institutes of Health: Grant/Research Support|Shionogi: Advisor/Consultant|The Health Resources & Services Administration: Grant/Research Support **Nicholas Van Hise, PharmD**, Ferring Pharmaceuticals Inc.: Advisor/Consultant|Ferring Pharmaceuticals Inc.: investigator **Glenn S. Tillotson, PhD**, Dynavax: Advisor/Consultant|Ferring Pharmaceuticals: Advisor/Consultant|Peggy Lillis Foundation: Honoraria|Spero Therapeutics: Advisor/Consultant

